# Study on the Shear Modulus Based Equivalent Homogenization Methods of Multi-Layer BCC Lattice Sandwich

**DOI:** 10.3390/ma15041341

**Published:** 2022-02-11

**Authors:** Wukun Zhang, Jian Zhao, Yonghua Tan, Yushan Gao, Jun Wang, Xiaoliang Geng

**Affiliations:** 1 Xi’an Aerospace Propulsion Institute, Xi’an 710100, China; casc_zhao@163.com (J.Z.); gao_yushan@163.com (Y.G.); junw83@163.com (J.W.); 2 National Key Laboratory of Science and Technology on Liquid Rocket Engines, Xi’an 710100, China; tanyhcasc@163.com; 3 Academy of Aerospace Propulsion Technology, Xi’an 710100, China; 4 School of Mechanics, Civil Engineering and Architecture, Northwestern Polytechnical University, Xi’an 710072, China; gengxiaoliang@nwpu.edu.cn

**Keywords:** multi-layer BCC lattice structures, homogenization, equivalent shear modulus, shear experiments, selective laser melting

## Abstract

In this paper, the shear modulus based equivalent homogenization methods of multi-layer BCC (body-centered cubic) lattice sandwich structures have been studied using analytical, experimental, and finite element methods. In the analytical approach, the multiple strut-deformation patterns were introduced in the derivations of the shear modulus based on Euler–Bernoulli beam theory and Timoshenko beam theory according to different boundary conditions. The analytical shear modulus of three types of rectangle shaped sandwich BCC lattice structures was derived. Finite element models of the BCC lattice structures by ANSYS were conducted to estimate the analytical solutions. Butterfly style sandwich BCC lattice structures were printed by SLM technology using 304 stainless steel (06Cr19Ni10), and corresponding shear experiments using modified Arcan Rig experimental devices were conducted to validate the analytical and numerical calculations. Good agreements were observed among the analytical, numerical, and experimental results.

## 1. Introduction

Lattice structures are periodic porous structures with many advantages, such as light weight, high strength, energy absorption, heat insulation, and heat dissipation [1]. Meanwhile, the large open space inside the lattice structures can also be filled with other materials or structures, which will also support the material-structure-performance integrated (MSFI) design [2]. Based on the various functions, lattice structures have been widely used in aerospace [3], medicine [4], and other fields.

Considerable investigations have focused on the shear mechanical properties of lattice structures. Xiong [5] and Dong [6] studied the shear properties of pyramid and octet lattice structures made of carbon fiber composites, respectively. Based on the energy method, Du [7] optimized lattice configurations with maximum shear stiffness. Feng et al. [8] conducted the shear and bending properties of hourglass lattice structures. They found that the specific shear strength was higher than that of pyramid structures. Zhang et al. [9] investigated the compression, shear, and bending properties of X-type lattice structures by theoretical and numerical simulations. Liu et al. [10] tested tensile and shear properties of star lattice structures and considered that the structures had large strain effects.

The traditional manufacturing methods of lattice structures include perforated metal sheet forming, snap fitting, investment casting, and so on. Additive manufacturing [11] (AM) technology is a new method to fabricate lattice structures. By means of layered discrete-laminated stacking, it has obvious advantages in manufacturing special cell configurations, hierarchy lattice structures [12], and plate lattice structures [13]. BCC lattices are bending-dominated lattice structures which are often used as structures in anti-impact and energy absorption. These body-centered configurations are also suitable for the manufacturing characteristics of AM technology. Therefore, BCC lattice structures fabricated by AM have been widely investigated by many scholars [14]. Tsopanos [15] studied manufacturing process parameters on the effects of axial compression properties on BCC lattice structures by laser selective laser melting (SLM). Gumruk [16] examined mechanical properties of BCC, BCCZ and F_2_BCC lattice cores under compression, shear, and tension-shear loadings fabricated by SLM technology using stainless steel. They found the effects of the geometry and relative density had a great influence on BCC lattice cores. Leary [17,18] investigated failure modes and absorption properties of BCC cores in various configurations fabricated by SLM technology using ALSi12Mg and Inconel 625. Li [19] conducted a series of compression tests of BCCZ manufactured by SLM technology using ALSi12Mg. Lee [20] studied the mechanical properties of BCC and FCC lattice structures under compression loadings and considered that Young’s modulus of BCC was higher than that of FCC under boundary restrained conditions. Lei [21] derived the effects of boundary conditions on the energy absorption properties of multilayer BCC and BCCZ lattice structures under compression loadings. The lattice cores were fabricated by SLM technology using ALSi12Mg. However, shear performance of BCC lattice sandwich structures under boundary constrained conditions have not been fully investigated in these experiments.

It is the premise of engineering design to master the mechanical properties of lattice structures. There are three kind of methods to study lattice structures in FEM (Finite element method), namely beam model, solid model, and homogenization model. The process of using refined models by beam and solid model can produce the mechanical properties of lattices correctly. However, it is inconvenient to use the two methods mentioned above to study lattices in engineering applications, for the amount of calculations and workload required. Therefore, the homogenization method [22,23,24] is necessary in studying the mechanical properties of lattice structures. A significant amount of research has been undertaken to study the modulus of BCC lattice structures by macro equivalent methods. Ptochos [25,26] derived the equivalent Young’s modulus and shear modulus of multi-layer BCC lattice cores. Liu et al. [27] investigated the equivalent Young’s modulus of multi-layer BCC lattice sandwich on the basis of “macro-single cell” deformation hypothesis. Yang et al. [28] considered that there were two typical deformation modes of the multi-layer BCC lattice sandwich in the process of compression loadings according to different boundary conditions, and derived the equivalent Young’s modulus of multi-layer sandwich BCC lattices by macro assembly method. Ushijima [29,30] believed that there were two typical deformation modes in the shear process of BCC lattice. In addition, the equivalent shear modulus of the two modes was studied by finite elements and theoretical analysis based on Euler–Bernoulli beam theory. However, the assembly method was induced by a large number of finite element statistical results. Meanwhile, this assumption has not been verified by experiments.

In this paper, a new macro equivalent analysis model is proposed to study the shear modulus of BCC lattice structures. Based on the deformation modes of three topological shapes of BCC lattice structures in shear loadings, two typical deformation patterns of BCC cores are conducted. The Timoshenko beam theory and Euler–Bernoulli beam theory are both applied to determine the shear modulus of the unit cells with different deformation patterns. Numerical simulation methods are also applied to investigate the deformation patterns by ANSYS. Two kinds of sandwich BCC lattices are printed by SLM technology with 304 stainless steel. The shear tests are also carried out using modified Arcan Rig experimental devices to validate the analytical model and the finite element model.

## 2. Shear Modulus Theory Model of Multi-Layer BCC Lattice Structures

### 2.1. Coordinate Transformation Method

**Figure 1:**
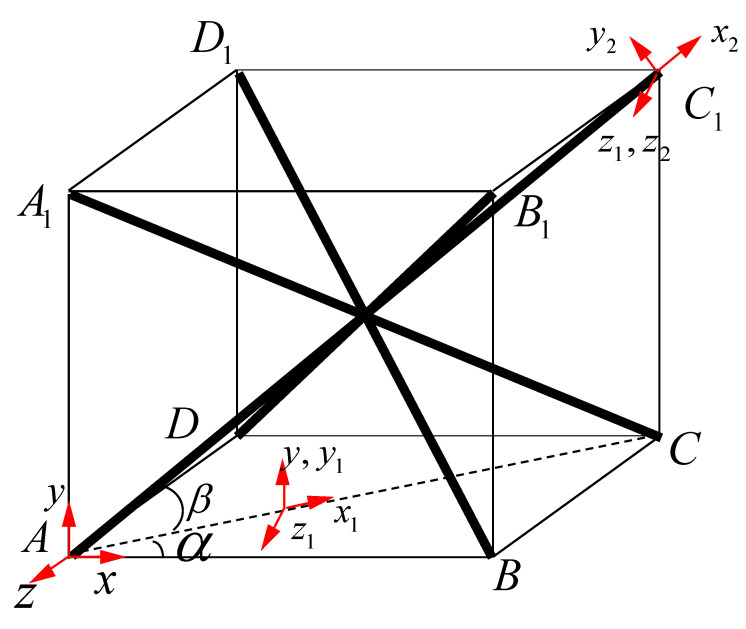
Process of coordinate system conversion.

A single cell of a BCC lattice structure is shown in Figure 1. The original coordinate system is xyz. After two rotations the coordinate system is $x_{2}y_{2}z_{2}$ According to the coordinate transformation method, the rotating coordinate system changes from the horizontal direction along x axis to the direction along the bar ($x_{2}$ axis). The first angle is α by rotation around the axis y and the second angle is β by rotation around the axis $z_{1}$.

According to the transformation formula of the coordinate rotation matrix, the transformation relation between coordinate matrix X in coordinate system xyz and coordinate matrix $X_{2}$ in coordinate system $x_{2}y_{2}z_{2}$ is:

(1) $$X_{2}=\Phi X$$

where Φ is an orthogonal matrix: $\Phi^{T}=\Phi^{-1}$

$$X_{2}=\left[\begin{array}{c} x_{2}\\ y_{2}\\ z_{2} \end{array}\right],X=\left[\begin{array}{c} x\\ y\\ z \end{array}\right],\Phi =\left[\begin{array}{ccc} \cos\alpha \cos\beta & \sin\beta & -\sin\alpha \cos\beta \\ -\cos\alpha \sin\beta & \cos\beta & \sin\alpha \cos\beta \\ \sin\alpha & 0 & \cos\alpha \end{array}\right]$$

**Figure 2:**
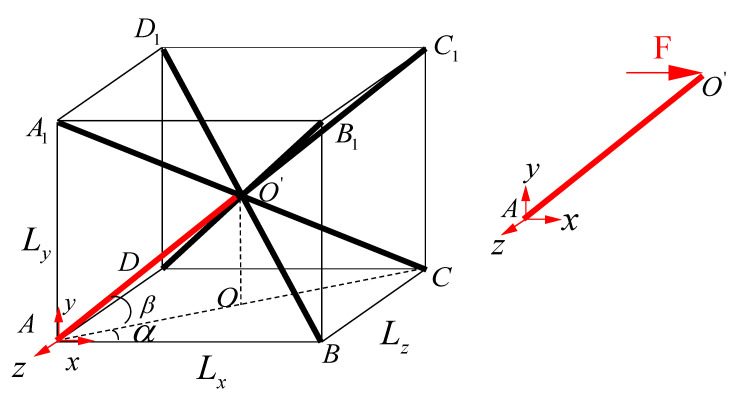
Homogenization equivalent method of BCC lattice structure.

As shown in Figure 2, since the BCC lattices are symmetrical during the shear loading process and the stress states of the four lower members are the same, it is reliable to choose a typical element to calculate the equivalent shear modulus-like bar $AO^{\prime }$.

For the red bar $AO^{\prime }$, the equivalent shear modulus of the force F acting on the plane xoz is:

(2) $$G=\frac{\tau_{xz}}{\gamma_{xz}}$$

where $\tau_{xz}$ is the in-plane shear stress in the plane xoz, $\gamma_{xz}$ is the in-plane shear strain in the plane xoz:

(3) $$\tau_{xz}=\frac{4F}{S_{ABCD}}$$

(4) $$\gamma_{xz}=\frac{2m}{L_{y}}$$

(5) $$\left\{\begin{array}{c} {\left(2l\right)}^{2}=L_{x}{}^{2}+L_{y}{}^{2}+L_{z}{}^{2}\\ \begin{array}{c} L_{x}=2l\cos\beta \cos\alpha \\ L_{z}=2l\cos\beta \sin\alpha \end{array}\\ L_{y}=2l\sin\beta \end{array}\right.$$

In Formulas (3)–(5), the side lengths of the equivalent hexahedron $ABCD-A^{\prime }B^{\prime }C^{\prime }D^{\prime }$ in Figure 2 are $L_{x}$, $L_{y}$ and $L_{z}$, respectively. The base area of the plane xoz is $S_{ABCD}$, and the displacement of point $O^{\prime }$ along the axis x is m. The length of the bar $AC_{1}$ is 2l, then the length of the bar $AO^{\prime }$ in Figure 2 is l.

The parameters of transition matrix Φ in Formula (1) can be determined by the following Formulas (6) and (7):

(6) $$\cos\alpha =\frac{L_{x}}{\sqrt{L_{x}{}^{2}+L_{z}{}^{2}}}$$

(7) $$\cos\beta =\frac{\sqrt{L_{x}{}^{2}+L_{z}{}^{2}}}{2l}$$

**Figure 3:**
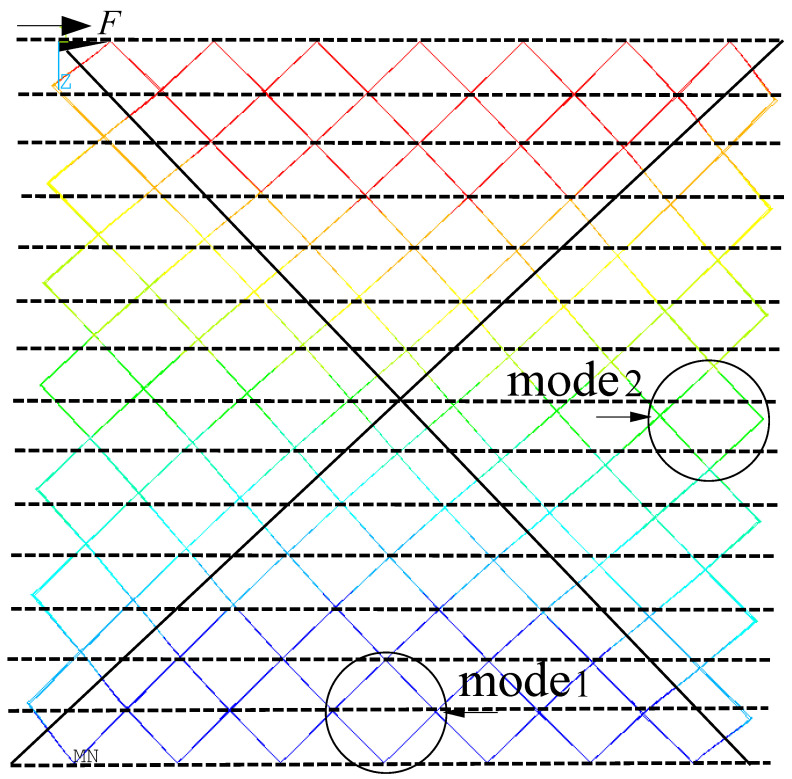
Deformation pattern of multi-layer BCC lattice structures.

In the common shear loadings of the BCC sandwich shown in Figure 3, the top nodes are coupling constrained degrees of freedom and apply shear force in the right direction, the bottom nodes are all fixed, and the left and right nodes are unconstrained. In this case, the bars of the multi-layer BCC lattice sandwich are dominated by two typical deformation modes and the whole model can be partitioned in four parts according to deformation. One is under the constrained boundary, as mode 1 shown in Figure 3, the other is under the unconstrained boundary, as mode 2 shows in Figure 3. In mode1, the nodes are in or very close to the black dotted line, so this mode can be seen as a constrained boundary. The bottom and top parts belong to mode1. In mode2, the nodes are away from the black dotted line, so this mode can be seen as unconstrained boundary. The left and right parts belong to mode2.

### 2.2. Equivalent Shear Modulus of Typical Element under Constrained Boundary

**Figure 4:**
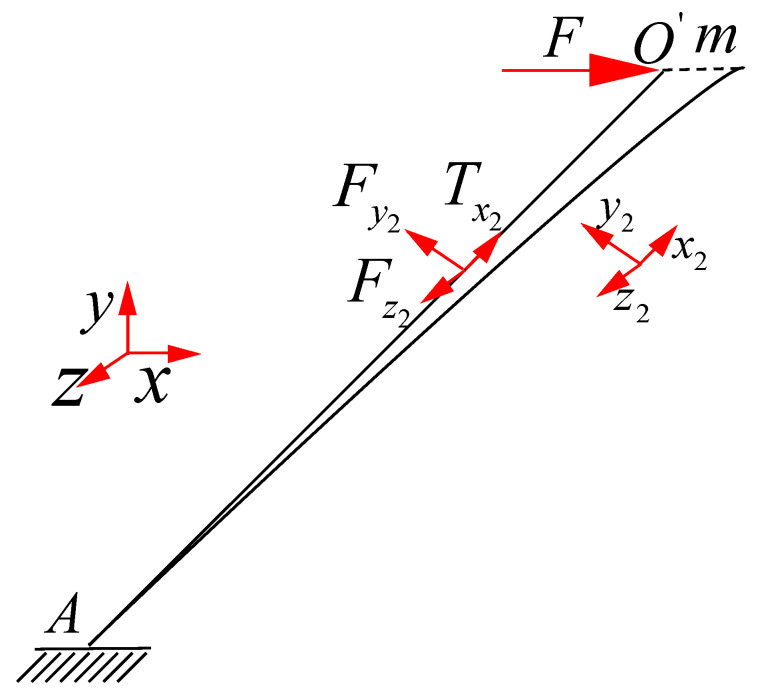
Typical element deformation mode under constraint boundary.

As shown in Figure 4, the first deformation pattern (mode1) is the typical element mode in the constrained boundary of the y direction. The force acting on the point $O^{\prime }$ along the axis x in the coordinate system xyz is F and the displacement along the direction y is zero. The deformation of the point $O^{\prime }$ in the coordinate system xyz is (m,0,0). The deformation of the point $O^{\prime }$ in the coordinate system $x_{2}y_{2}z_{2}$ is $\left(\delta_{x_{2}},\delta_{y_{2}},\delta_{z_{2}}\right)$, which can be calculated by Formulas (8)–(10) using the coordinate transformation method:

(8) $$\delta_{x_{2}}=m\cos\alpha \cos\beta$$

(9) $$\delta_{y_{2}}=-m\cos\alpha \sin\beta$$

(10) $$\delta_{z_{2}}=m\sin\alpha$$

The acting force of point $O^{\prime }$ in the coordinate system $x_{2}y_{2}z_{2}$ is $\left(T_{x_{2}},F_{y_{2}},F_{z_{2}}\right)$. The relationship between force and displacement can be calculated as Formula (11)–(13):

(11) $$\delta_{x_{2}}=\frac{T_{x_{2}}l}{E_{s}A}$$

(12) $$\delta_{y_{2}}=\left\{\begin{array}{l} \frac{F_{y_{2}}l^{3}}{12E_{s}I_{y}{}^{e}}+\frac{F_{y_{2}}l}{E_{s}\pi r^{2}}\frac{7+6\nu_{s}}{3}\;\mathrm{Tim}\;\\ \frac{F_{y_{2}}l^{3}}{12E_{s}I_{y}{}^{e}}\;E-B\; \end{array}\right.$$

(13) $$\delta_{z_{2}}=\frac{F_{z_{2}}l^{3}}{3E_{s}I_{z}{}^{e}}$$

where r is the radius of the bar. A is the cross-sectional area of the bar. $I_{y}^{e}$ and $I_{z}^{e}$ are the second moment of inertia of the strut circular cross section along the directions y and z, respectively. $A=\pi r^{2}$. $I_{y}{}^{e}=I_{z}{}^{e}=\frac{\pi r^{4}}{4}$. $E_{s}$ is the young’s modulus of the material. $\nu_{s}$ is the Poisson’s ratio. In Formula (12), Tim denotes the load-displacement relation based on Timoshenko beam theory, while E-B denotes the load-displacement relation based on Euler–Bernoulli beam theory.

According to Formula (1), the relationship between the force F and the acting force $\left(T_{x_{2}},F_{y_{2}},F_{z_{2}}\right)$ can be obtained as Formula (14):

(14) $$F=\cos\alpha \cos\beta \cdot T_{x}{}_{{}_{2}}-\cos\alpha \sin\beta \cdot F_{y}{}_{{}_{2}}+\sin\alpha \cdot F_{z}{}_{{}_{2}}$$

According to Formulas (2)–(14), the equivalent shear modulus $G_{1}$ under constrained boundary can be calculated as Formula (15):

(15) $$\mathrm{Euler}-\mathrm{Bernoulli}\;\mathrm{solution}:\;\;G_{1}=\frac{\pi E_{s}}{4}\frac{\sin\beta }{\cos\alpha \cos^{2}\beta \sin\alpha }{\left(\frac{r}{l}\right)}^{2}\cdot [4\cos^{2}\alpha \cos^{2}\beta +3{\left(\frac{r}{l}\right)}^{2}\left(4\cos^{2}\alpha \sin^{2}\beta +\sin^{2}\alpha \right)]\;\mathrm{Timoshenko}\;\mathrm{solution}:\;\;G_{1}=\frac{\pi E_{s}}{4}\frac{\sin\beta \pi E_{s}}{\cos\alpha \cos^{2}\beta \sin\alpha }{\left(\frac{r}{l}\right)}^{2}\cdot [4\cos^{2}\alpha \cos^{2}\beta +\frac{12\cos^{2}\alpha \sin^{2}\beta }{{\left(\frac{l}{r}\right)}^{2}+7+6v_{s}}+3\sin^{2}\alpha {\left(\frac{r}{l}\right)}^{2}]$$

Especially, when the BCC lattice cell is a cube structure, we can find $L_{x}=L_{y}=L_{z}$: $\cos\alpha =\frac{1}{\sqrt{2}}$, $\cos\beta =\frac{\sqrt{2}}{\sqrt{3}}$.

In this case, the equivalent shear modulus $G_{1}$ under constrained boundaries can be calculated as Formula (16):

(16) $$G_{1}=\left\{\begin{array}{l} \frac{\sqrt{3}\pi E_{s}r^{2}}{l^{2}}[\frac{1}{3}+\frac{1}{2(\frac{l^{2}}{r^{2}}+7+6\nu_{s})}+\frac{3}{8}\frac{r^{2}}{l^{2}}]\;\mathrm{Tim}\\ \frac{\sqrt{3}\pi E_{s}r^{2}}{l^{2}}[\frac{1}{3}+\frac{7}{8}\frac{r^{2}}{l^{2}}]\;E-B \end{array}\right.$$

### 2.3. Equivalent Shear Modulus of Typical Element under Unconstrained Boundary

**Figure 5:**
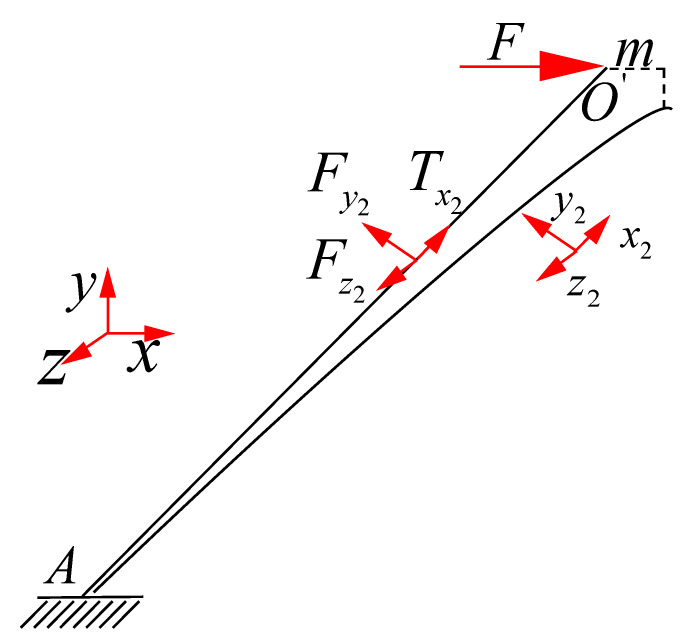
Typical element deformation mode under free boundary.

The second deformation pattern (mode2) is the typical element mode under the unconstrained boundary of the y direction. As shown in Figure 5, in the coordinate system xyz, the displacement of the point $O^{\prime }$ along the direction x is m. The point $O^{\prime }$ along the direction y also has the displacement, which is different from mode1. However, in the coordinate system $x_{2}y_{2}z_{2}$, the displacement of the point $O^{\prime }$ along the direction $x_{2}$ is zero.

According to the Formula (1), the displacement of the point $O^{\prime }$ in the coordinate system xyz is $\left(m,0,-\frac{\cos\alpha \cos\beta }{\sin\beta }m\right)$. The displacement of the point $O^{\prime }$ in the coordinate system $x_{2}y_{2}z_{2}$ is $\left(0,-\frac{\cos\alpha }{\sin\beta }m,m\sin\alpha \right)$:

(17) $$\delta_{x_{2}}=0$$

(18) $$\delta_{y_{2}}=-\frac{\cos\alpha }{\sin\beta }m$$

(19) $$\delta_{z_{2}}=m\sin\alpha$$

(20) $$\delta_{z_{2}}=\left\{\begin{array}{l} \frac{F_{z_{2}}l^{3}}{12E_{s}I_{z}{}^{e}}+\frac{F_{z_{2}}l}{E_{s}\pi r^{2}}\frac{7+6\nu_{s}}{3}\;\mathrm{Tim}\\ \frac{F_{z_{2}}l^{3}}{12E_{s}I_{z}{}^{e}}\;E-B \end{array}\right.$$

(21) $$\delta_{y_{2}}=\frac{F_{y_{2}}l^{3}}{3E_{s}I_{y}{}^{e}}$$

where the deformation of the point $O^{\prime }$ in the coordinate system $x_{2}y_{2}z_{2}$ is $\left(\delta_{x_{2}},\delta_{y_{2}},\delta_{z_{2}}\right)$.

Projection F to the axis x, according to the Formula (1):

(22) $$F=-\cos\alpha \sin\beta \cdot F_{y_{2}}+\sin\alpha F_{z_{2}}$$

According to Formulas (2)–(7) and (17)–(22), the equivalent shear modulus $G_{2}$ under unconstrained boundary can be calculated as Formula (23):

(23) $$\mathrm{Euler}-\mathrm{Bernoulli}\;\mathrm{solution}:\;\;G_{2}=\frac{3\pi E_{s}}{4}{\left(\frac{r}{l}\right)}^{4}(\frac{\cos^{2}\alpha +4\sin^{2}\alpha }{\sin\alpha \cos\alpha \cos^{2}\beta })\sin\beta \;\mathrm{Timoshenko}\;\mathrm{solution}:\;\;G_{2}=\frac{3\pi \sin\beta E_{s}}{4}{\left(\frac{r}{l}\right)}^{2}[\frac{\cos\alpha }{\sin\alpha \cos^{2}\beta }{\left(\frac{r}{l}\right)}^{2}+\frac{4\sin\alpha }{\cos\alpha \cos^{2}\beta }\cdot \frac{1}{{\left(\frac{l}{r}\right)}^{2}+7+6v_{s}}]$$

In particular, when the BCC lattice cell is a cube structure, we can find $L_{x}=L_{y}=L_{z}$, $\cos\alpha =\frac{1}{\sqrt{2}}$, $\cos\beta =\frac{\sqrt{2}}{\sqrt{3}}$. In this case, the equivalent shear modulus $G_{2}$ under free boundaries can be calculated as Formula (24):

(24) $$G_{2}=\left\{\begin{array}{l} \frac{3\sqrt{3}\pi E_{s}r^{2}}{l^{2}}[\frac{1}{2(\frac{l^{2}}{r^{2}}+7+6\nu_{s})}+\frac{1}{8}\frac{r^{2}}{l^{2}}]\;\mathrm{Tim}\\ \frac{15\sqrt{3}\pi E_{s}r^{4}}{8l^{4}}\;E-B \end{array}\right.$$

### 2.4. BCC Lattice Shear Modulus Assembly under Two Deformations

**Figure 6:**
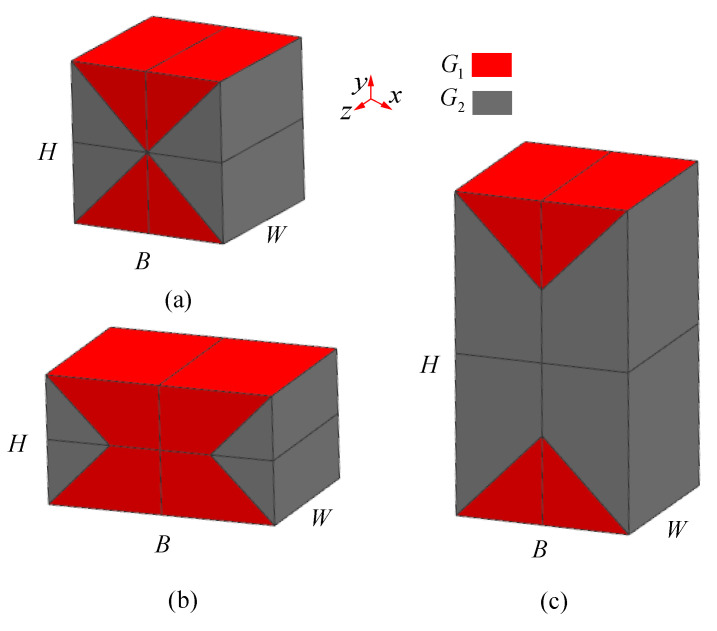
Typical macro-structural form (**a**) H = B; (**b**) H < B; (**c**) H > B.

As shown in Figure 6, the macro representation of a typical rectangle shaped lattice sandwich structure has three topological shapes: (a) H = B; (b) H < B; (c) H > B. H is the height of cores, B is the length of cores and W is the width of cores. According to the deformation mode in Figure 3, the lattice structure of multi-layer BCC can be divided into four parts according to the macro deformations which are separated along the diagonal shearing plane. In the case of H = B, the plane intersects the center line, as shown in Figure 6a. In the case of H < B, the plane intersects in the horizontal center plane, as shown in Figure 6b. In the case of H < B, the plane intersects in the vertical center plane, as shown in Figure 6c. The macro-shear modulus assemblies of these three patterns are analyzed respectively as follows.

#### 2.4.1. H = B

**Figure 7:**
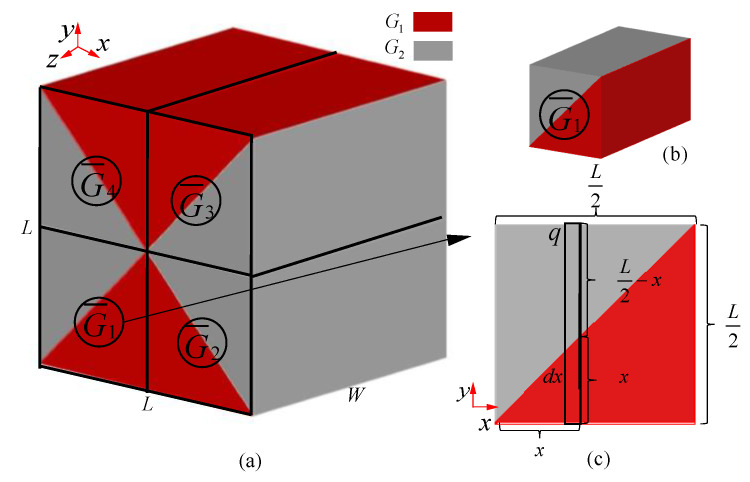
Group concept of macro equivalent shear modulus for multi-layer BCC lattice structures (H = B): (**a**) the whole model; (**b**) the first part; (**c**) the representative unit calculation in *x*-*y* plane.

When H = B = L, $\bar{G}$ is the equivalent shear modulus of the whole model, in which the equivalent shear modulus of the red part is $G_{1}$, and the shear modulus of the gray part is $G_{2}$. The whole model is divided into four parts according to the position, and the macroscopic shear modulus of the four parts is $\bar{G_{1}},\bar{G_{2}},\bar{G_{3}},\bar{G_{4}}$, respectively. The macro equivalent shear modulus of the first part in Figure 7a is shown in Figure 7b. Since the shear modulus along the axis z is the same, it can be analyzed only in plane xy, as shown in Figure 7c.

(25) $${\bar{G}}_{1}=\frac{F_{1}L/2}{\delta_{1}S_{1}}$$

where $F_{1}$ is the shearing force subjected to the uniform body, $\delta_{1}$ is the shear deformation and $S_{1}$ is the area of the shear plane. Thus, $S_{1}=LW/2$:

(26) $$F_{1}=\int_{0}^{L/2}qWdx$$

where q is the shearing force per unit area. Consequently, qWdx is the shearing force of the segment dx.

Total deformation of segment dx can be calculated as Formula (27):

(27) $$\delta_{dx}=\frac{q(L/2-x)}{G_{2}}+\frac{qx}{G_{1}}$$

Because in the shear deformation of each segment dx is equal, Formulas (28) and (29) can be calculated:

(28) $$\delta_{dx}=\delta_{1}$$

(29) $$\frac{\partial \delta_{1}}{\partial x}=0$$

According to Formulas (27)–(29):

(30) $$q=\frac{1}{\frac{L}{2}G_{1}+(G_{2}-G_{1})x}$$

Take Formula (30) in Formula (26):

(31) $$\delta_{1}=\frac{1}{G_{1}G_{2}}$$

According to Formulas (25)–(31)

The equivalent shear modulus of the first part can be calculated as Formula (32):

(32) $${\bar{G}}_{1}=\frac{\ln(G_{2}/G_{1})G_{1}G_{2}}{G_{2}-G_{1}}$$

In the same way, the equivalent shear modulus of the four parts can be calculated as Formula (33):

(33) $${\bar{G}}_{1}={\bar{G}}_{2}={\bar{G}}_{3}={\bar{G}}_{4}$$

Consequently, the equivalent shear modulus of the whole part is $\bar{G}=\frac{\ln(G_{2}/G_{1})G_{1}G_{2}}{G_{2}-G_{1}}$.

#### 2.4.2. H < B

**Figure 8:**
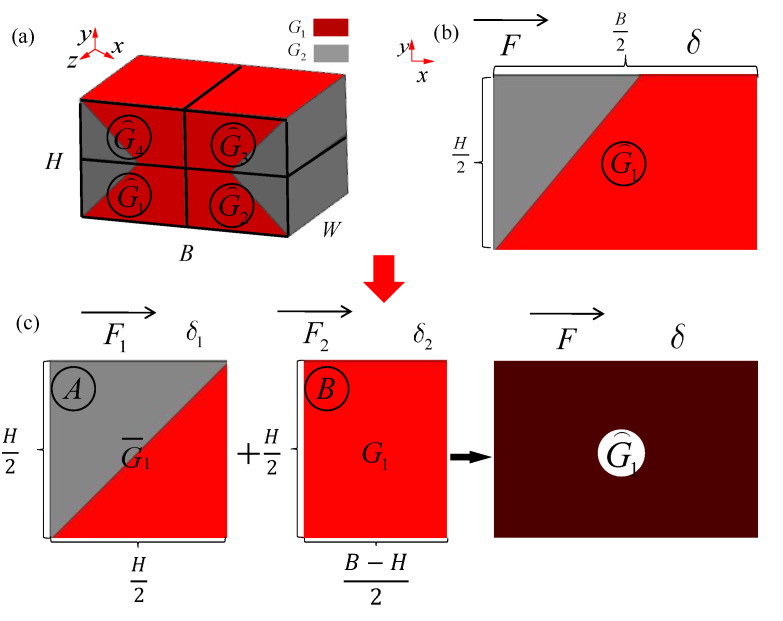
Group concept of macro equivalent shear modulus for multi-layer BCC lattice structures (H < B): (**a**) the whole model; (**b**) the first part; (**c**) the representative unit calculation in *x*-*y* plane.

As shown in Figure 8a, in the case of H < B, $\overset{⌢}{G}$ is the equivalent shear modulus of the whole model. The whole model is also divided into four parts according to the position, and the macro shear modulus of the four parts are ${\overset{⌢}{G}}_{1},{\overset{⌢}{G}}_{2},{\overset{⌢}{G}}_{3},{\overset{⌢}{G}}_{4}$, respectively. The macro equivalent shear modulus of the first part in Figure 8a is shown in Figure 8b. The shear modulus ${\overset{⌢}{G}}_{1}$ in Figure 8b can be easily divided into two parts of which shear modulus are already known in 2.4.1. One is body A, and the shear modulus is ${\bar{G}}_{1}$, the other is body B, and the shear modulus is $G_{1}$. Therefore, the shear modulus of the first part can be analyzed as shown in Figure 8c.

(34) $${\overset{⌢}{G}}_{1}=\frac{FH/2}{\delta S}$$

In the Formula (34), F is the shearing force in Figure 8b. δ is the shear deformation. S is the area of the shear plane. Thus, S=BW/2:

(35) $$\left\{\begin{array}{c} \delta_{1}=\frac{F_{1}H/2}{{\bar{G}}_{1}S_{1}}\\ \delta_{2}=\frac{F_{2}H/2}{G_{1}S_{2}} \end{array}\right.$$

(36) $$\left\{\begin{array}{c} F=F_{1}+F_{2}\\ \delta =\delta_{1}=\delta_{2}\\ S=S_{1}+S_{2} \end{array}\right.$$

(37) $$\left\{\begin{array}{c} S_{1}=\frac{HW}{2}\\ S_{2}=\frac{(B-H)W}{2} \end{array}\right.$$

In Formulas (35)–(37), $\delta_{1}$, $F_{1}$ and $S_{1}$ are the shear deformation, shearing force and shear area of body A, respectively, while $\delta_{2}$, $F_{2}$ and $S_{2}$ are the shear deformation, shearing force and shear area of body B. respectively, in Figure 8c.

According to Formulas (34)–(37), the equivalent shear modulus of the first part can be calculated as Formula (38):

(38) $${\overset{⌢}{G}}_{1}=\frac{(B-H)G_{1}+H{\bar{G}}_{1}}{B}$$

In the same way, the equivalent shear modulus of the four parts can be calculated as Formula (39):

(39) $${\overset{⌢}{G}}_{1}={\overset{⌢}{G}}_{2}={\overset{⌢}{G}}_{3}={\overset{⌢}{G}}_{4}$$

Consequently, the equivalent shear modulus of the whole part is $\overset{⌢}{G}=\frac{(B-H)G_{1}+H\bar{G}}{B}$.

Especially, when the value of B−H reach zero, the $\overset{⌢}{G}$ will also move to $\bar{G}$.

#### 2.4.3. H > B

**Figure 9:**
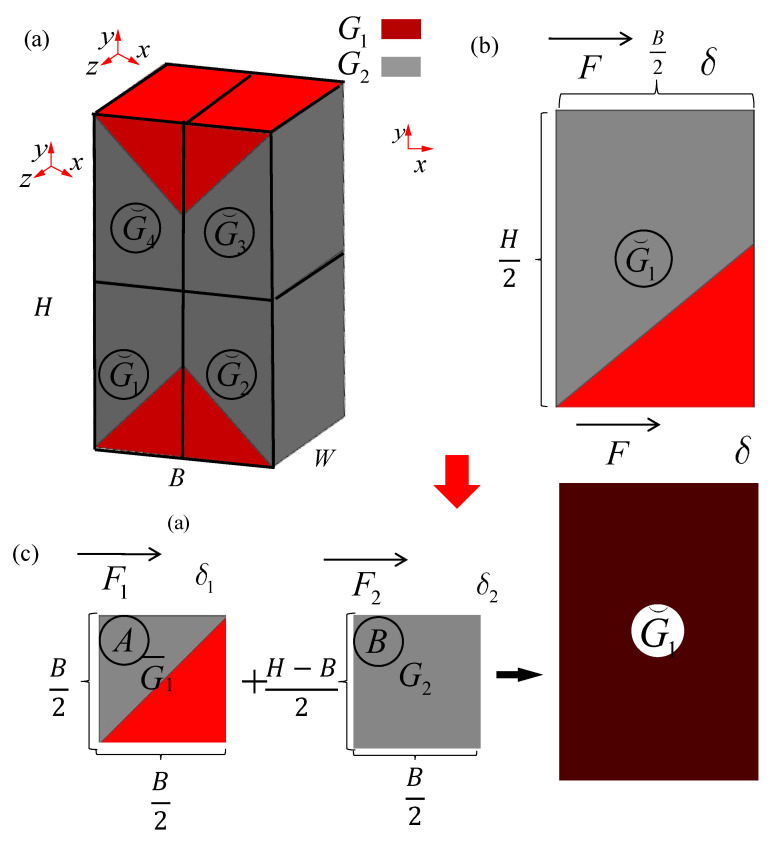
Group concept of macro equivalent shear modulus for multi-layer BCC lattice structures (H > B): (**a**) the whole model; (**b**) the first part; (**c**) the representative unit calculation in *x*-*y* plane.

In the case of H > B, $\overset{⌣}{G}$ is the equivalent shear modulus of the whole model. The whole model is divided into four parts according to the position, and the macroscopic shear modulus of the four parts are ${\overset{⌣}{G}}_{1},{\overset{⌣}{G}}_{2},{\overset{⌣}{G}}_{3},{\overset{⌣}{G}}_{4}$, respectively. The macroscopic equivalent shear modulus of the first part in Figure 9a is shown in Figure 9b. The shear modulus ${\overset{⌣}{G}}_{1}$ of Figure 9b can be easily divided in two parts already known. One is body A, of which shear modulus is ${\bar{G}}_{1}$, the other is body B, of which shear modulus is $G_{2}$. Therefore, the shear modulus of the first part can be analyzed as shown in Figure 9c:

(40) $${\overset{⌣}{G}}_{1}=\frac{FH/2}{\delta S}$$

In Formula (40), F is the shearing force in Figure 9b. δ is the shearing deformation. S is the area of the shear plane. Thus, S=BW/2.

In Formulas (41)–(44), $\delta_{1}$, $F_{1}$ and $S_{1}$ are the shear deformation, shearing force and shear area of body A, respectively, while $\delta_{2}$, $F_{2}$ and $S_{2}$ are the shear deformation, shearing force and shear area of body B, respectively, in Figure 9c.

(41) $$\left\{\begin{array}{c} \delta_{1}=\frac{F_{1}(H-B)/2}{{\bar{G}}_{1}S_{1}}\\ \delta_{2}=\frac{F_{2}B/2}{G_{1}S_{2}} \end{array}\right.$$

(42) $$\left\{\begin{array}{c} F=F_{1}=F_{2}\\ \delta =\delta_{1}+\delta_{2}\\ S=S_{1}=S_{2} \end{array}\right.$$

(43) $$\left\{\begin{array}{c} \delta_{1}=\frac{F_{1}H/2}{\delta_{1}S_{1}}\\ \delta_{2}=\frac{F_{2}H/2}{\delta_{2}S_{2}} \end{array}\right.$$

(44) $$S_{1}=S_{2}=\frac{HW}{2}$$

The equivalent shear modulus of the first part can be calculated as Formula (45):

(45) $${\overset{⌣}{G}}_{1}=\frac{H}{(H-B)/G_{2}+B/{\bar{G}}_{1}}$$

In the same way, the equivalent shear modulus of the whole part is:$\overset{⌣}{G}=\frac{H}{(H-B)/G_{2}+B/\bar{G}}$.

Especially, when the value of H−B reach zero, $\overset{⌢}{G}$ will also move to $\bar{G}$.

## 3. Experimental and Numerical Simulation Validations

**Figure 10:**
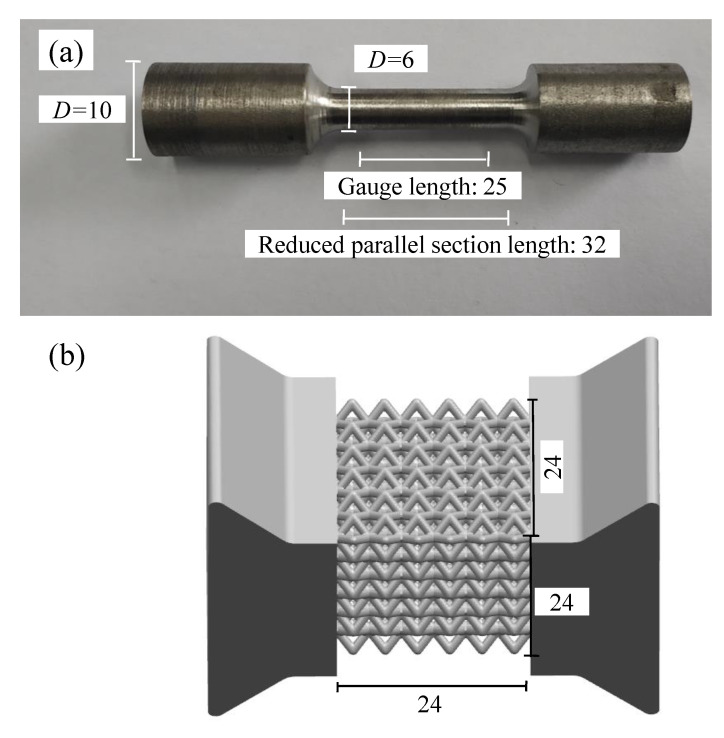
Basic size of test sample: (**a**) dumbbell-shaped specimen; (**b**) butterfly shaped specimen.

**Figure 11:**
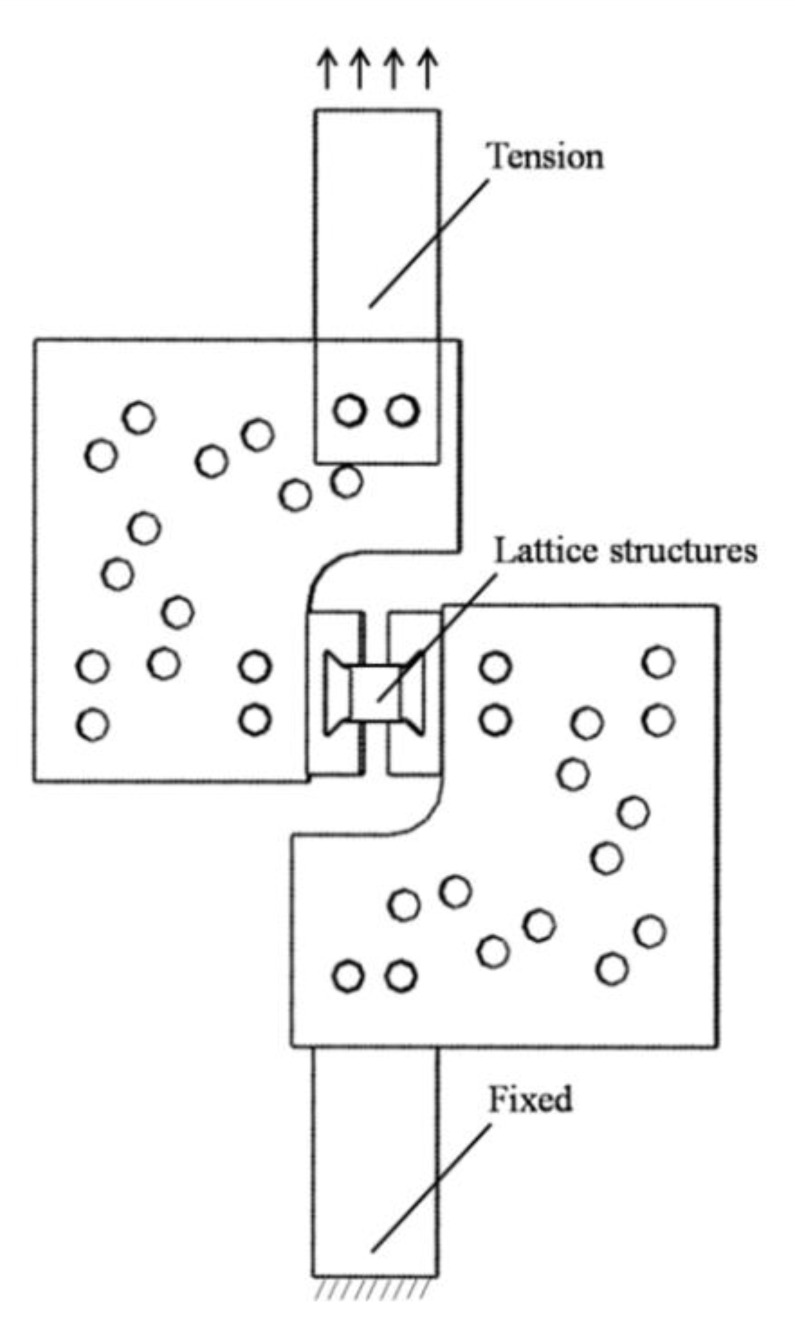
Principle of lattice structures shear test.

**Figure 12:**
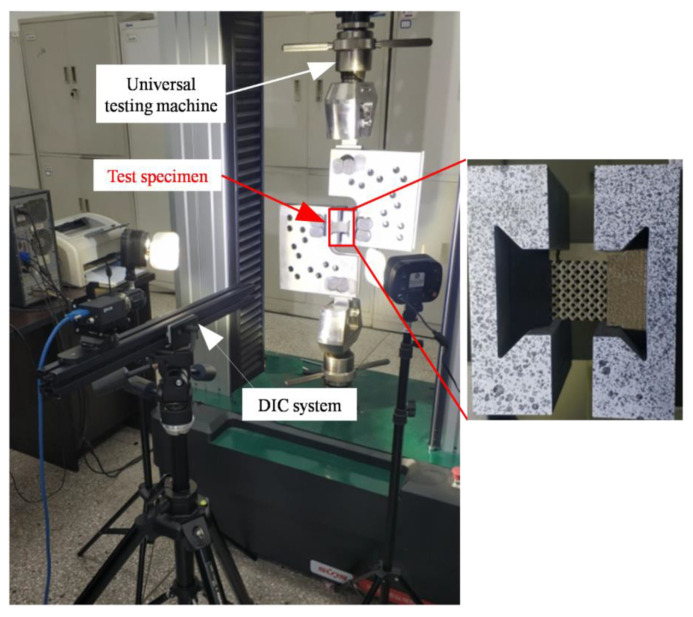
Loading process of test simple.

The multi-layer BCC lattice sandwich panel was manufactured by SLM technology. The material was 304 stainless steel (06Cr19Ni10). The material compositions are shown in Table 1. The printing machine was RENISHAW AM-400. The printing parameters are shown in Table 2. The basic size is shown in Figure 10. Figure 10a shows the printed dumbbell-shaped tensile standard specimen to test Young’s modulus according to ASTM standard E8/E8M-21. The diameter of the marked section is 6 mm, and the gauge length is 25 mm. Figure 10b shows the model size of the constrained boundary lattice structure test piece. The shape of the test piece is butterfly, which is convenient to be fixed with the fixture device. The cell size of the BCC cores is 4 mm × 4 mm × 4 mm, and the cell number is 6 × 6 × 6. The designed values of the rod’s slenderness ratio (r/l) are 0.145 and 0.175. Shear test was carried out by modified Rig experimental devices. The fixture shear test principle is shown in Figure 11. The experiments were carried out by a UTM5105 100 kN electronic universal testing machine using a uni-axial tensile loading. The shear loading process of the test piece is shown in Figure 12. The deformation of the lattice structure was measured and recorded using the extensometer and the non-contact measuring DIC device. The loading rate is 2 mm/min.

**Figure 13:**
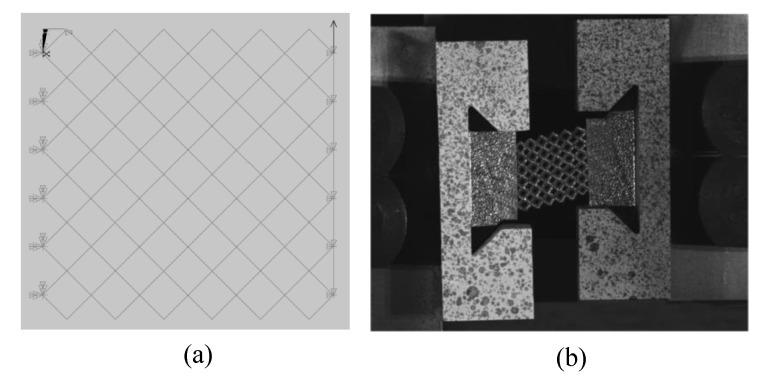
Deformation boundary conditions of the sandwich BCC lattice: (**a**) FE model; (**b**) sample.

The Young’s modulus of constitutive material is 202 GPa by uniaxial tensile loading tests. The Poisson’s ratio is 0.34. The finite element boundary condition is shown in Figure 13a using the commercial software ANSYS^®^ (ANSYS, Inc., Pittsburgh, PA, USA). The boundary condition was the fixed support of nodes on the left surface, and the nodes on the right surface were coupled with all degrees of freedom. Force along the negative x direction was applied to one of the nodes at the right surface. The lattice structures were modeled by beam188 beam element. In addition, the testing boundary conditions are shown in Figure 13b.

The shear modulus of finite element calculation and theoretical calculation is compared in Table 3. The shear modulus was calculated by Euler–Bernoulli beam theory. The whole length of cores is 40 mm, the number of single cells is 7, 10, and 13, and the lattice slenderness ratio is 0.13, 0.14, and 0.15, respectively. The results show that the relative error between the equivalent shear modulus calculated by FEM and analytical method is small. The maximum relative error is 7.55%, and the average relative error is 3.53%. The results indicate that the theoretical model can be applied to different numbers of lattice structures.

**Figure 14:**
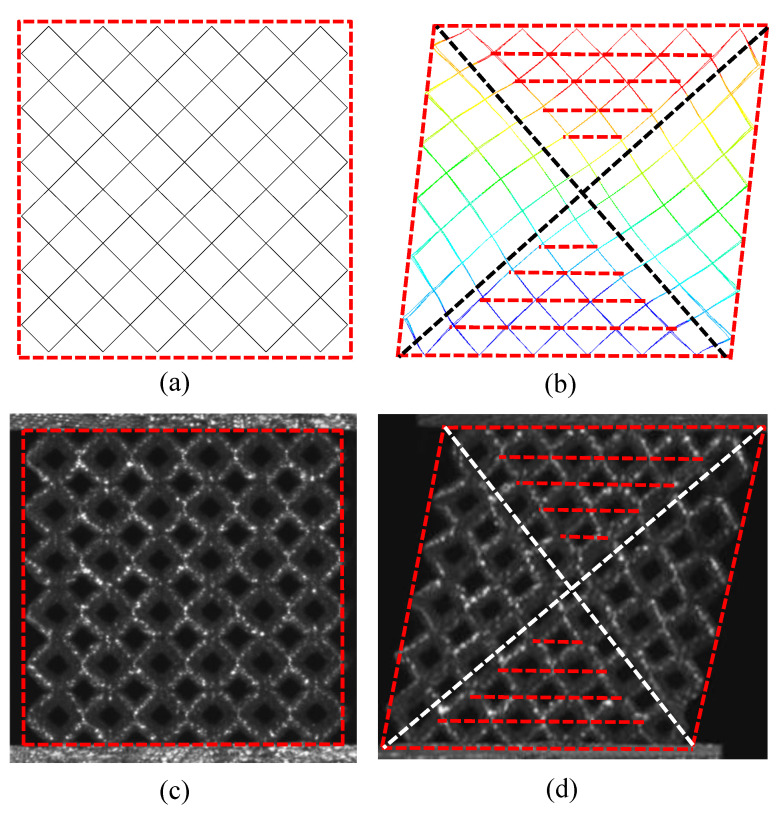
Deformation patterns of the sandwich BCC lattice: (**a**) initial FE model; (**b**) deformation of FE model; (**c**) initial state of sample; (**d**) deformed sample.

The deformation shapes of BCC lattice in experiments and simulations are compared in Figure 14. Figure 14a,c shows the original mode under unloaded conditions, and Figure 14b,d shows the deformation mode during shear loading. It can be seen that the deformation of each member in the shear process is in good agreement with that of the simulation, and the shape of the whole core is similar to a parallelogram. The element members exhibit a different degree of bending deformation, in which the bending deformation close to the unconstrained boundary is more obvious. Four planes in red color are used to depict the boundary envelope of the member under shear forces and the lattice structures are divided into four zones by the boundary of diagonal inclined planes in white color. In the left and right diagonal tri-prism regions, the deformation is approximately translational mode, and each cell element is similar to mode1 in Figure 3, where the nodes are in or very close to the red dotted line. Meanwhile, in the left and right diagonal tri-prism regions, the deformation is approximately the combination of translational and rotational mode, and the deformation of each cell element is similar to mode2 in Figure 3. The similar deformation shapes due to boundary effects of this lattice sandwich structures under loadings were also reported in [28,29,30].

The experimental results of shear modulus are compared with numerical simulation and theoretical analysis in Table 4. As can be seen from Table 4, the relative error of shear modulus of multi-layer BCC lattice structure obtained by experiments, theoretical calculation and numerical simulation is small. The maximum relative error between theoretical and experimental results is 4.6%, the average relative error of that is 2.78%. The maximum relative error between numerical and experimental results is 6.84%, and the average relative error of that is 4.86%. The good agreement of theoretical, numerical, and experimental data verifies the accuracy of the theoretical model and the numerical simulation model.

**Figure 15:**
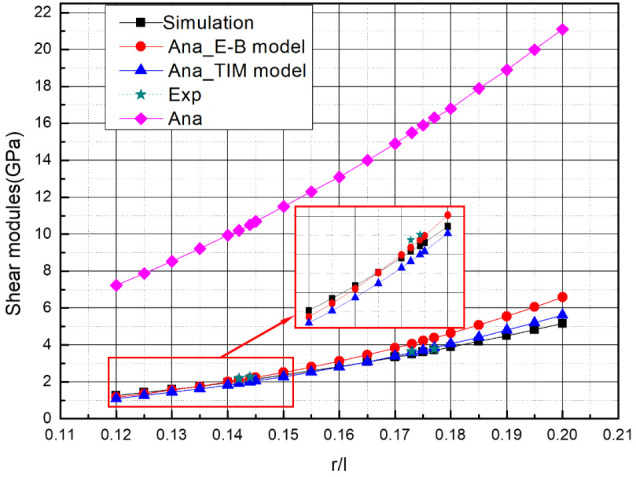
The curve of the shear modulus versus the slenderness ratio (slenderness ratio (r/l) = 0.12~0.2) of cubic sandwich BCC lattice structures.

As shown in Figure 15, the theoretical equivalent shear modulus using Euler–Bernoulli beam theory, the theoretical equivalent shear modulus using Timoshenko beam theory, the equivalent shear modulus of multi-layer BCC obtained by finite element calculations and experimental results, and the theoretical equivalent shear modulus by [26] are all compared together where the cell-element slenderness ratio (r/l) is 0.12~0.2. A good agreement between the analytical, experimental, and numerical results is observed. When the slenderness ratio (r/l) is 0.12~0.15, the analytical models using two beam theories are both close to numerical results and experimental results, while using the Euler–Bernoulli beam theory is nearer to numerical results and experimental results. However, when the slenderness ratio (r/l) is in 0.15~0.2, the analytical model using Timoshenko beam theory is closer to numerical results. The experimental results also indicate this phenomenon. However, the deformation shapes of members with different slenderness ratios are similar. Therefore, with the increase in slenderness, the bar thickens gradually, and the deformation of members conforms to the assumption of Timoshenko beam theory. In general, the theoretical equivalent shear modulus using Timoshenko beam theory is recommended for engineering applications in the whole range of slenderness ratio (r/l) .

**Figure 16:**
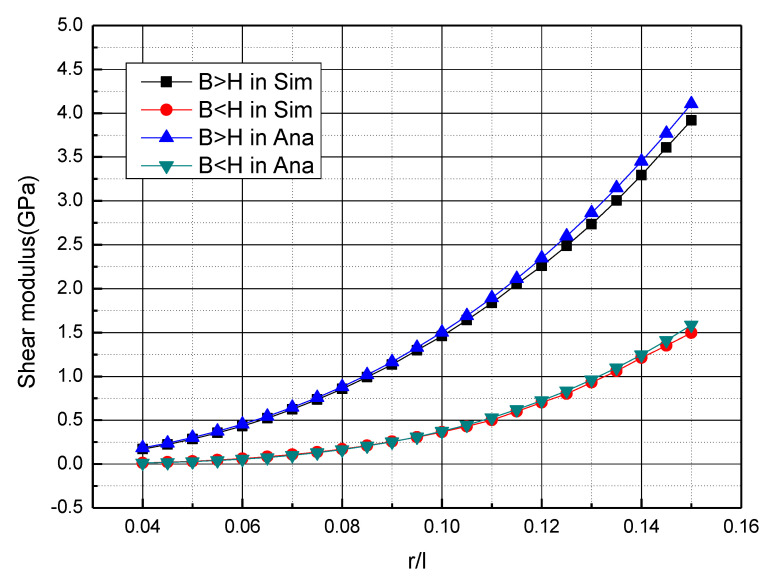
The curve of the shear modulus versus the slenderness ratio (r/l) of cubic sandwich BCC lattice structures of B > H and B < H.

As is shown in Figure 16, the theoretical analysis using Timoshenko beam theory and numerical simulation of shear modulus versus the slenderness ratio (r/l) to 0.04~0.16 for H > B and H < B are compared. Each single cell size is 4 mm × 4 mm × 4 mm. For H > B, the number of cells in H direction is 7 and the number of cells in B direction is 5, so H = 28 mm, B = 20 mm. As for H < B, the number of cells in H direction is 5 and the number of cells in B direction is 7, so H = 20 mm, B = 28 mm. It can be seen that the shear modulus of H > B and H < B is nearly the same when the slenderness ratio is small. When the slenderness ratio (r/l) increases, the shear modulus of both cases increases, but the shear modulus of H < B increases faster. The great agreement of theory and numerical simulation verifies the accuracy of the theoretical model under H > B and H < B.

## 4. Conclusions

In this paper, the macro equivalent shear modulus of three types of rectangle shaped sandwich BCC lattices structures with multi-layer boundary conditions is studied by theoretical, numerical, and experimental methods. In the theoretical part, the Euler–Bernoulli beam theory and Timoshenko beam theory are both used to model the shear modulus of BCC lattice structures. Different slenderness ratio lattices are manufactured by 304 stainless steel as the constitutive material. Shear tests are also conducted using modified Arcan Rig experimental devices. The main conclusions are as follows:

(1) The deformation mode of a multi-layer BCC lattice sandwich structure under shear loading is indicated by two typical deformation modes at the macroscopic scale partitioned under a diagonal shear plane. One is the constrained boundary, and the other is unconstrained boundary. Different shear boundary conditions lead to different shear deformation behaviors.
(2) The macro equivalent shear modulus theoretical model of multi-layer BCC lattice structures presented in this paper is in good agreement with the experimental and numerical simulation results, and can well reflect the macro shear mechanical properties.
(3) The deformation features of different BCC lattice member exhibit different degrees of bending under shear loadings. When the slenderness ratio (r/l)of the member increases gradually, the deformation of the member changes from the bending deformation mode based on the assumption of Euler–Bernoulli beam to the shear deformation mode based on the assumption of Timoshenko beam. When the slenderness ratio (r/l) increases, the shear modulus of both cases (H = B, H < B, H > B) increases, but the shear modulus of H < B increases faster. In general, the theoretical equivalent shear modulus using Timoshenko beam theory is recommended for multilayer BCC lattices in the whole range of the slenderness ratio (r/l) for convenience in engineering applications.

## Figures and Tables

**Table 1:** Chemical component of material table, wt%.

Element	C	Cr	Ni	Si	Mn
Values	≤0.08	18~20	8~11	≤1	≤2
Element	P	S	N	O	Fe
Values	≤0.045	≤0.03	≤0.25	≤0.1	other

**Table 2:** Printing process parameters.

Layer thickness	0.05 mm
Phase angle	67°
Laser power	250 w
Scan interval	0.11 mm
Scan speed	800 mm/s
Metallic powder	304 stainless steel

**Table 3:** Finite Element and Theoretical Derivation Calculation.

No	Cell Number	r/l	Simulation of Shear Modulus/GPa	Analysis of G1/GPa	Analysis of G2/GPa	Analysis of Shear Modulus/GPa	Error
1	7 × 7 × 7	0.13	1.61	6.46	0.59	1.55	3.83%
2	10 × 10 × 10	0.13	1.66				6.31%
3	13 × 13 × 13	0.13	1.68				7.55%
4	7 × 7 × 7	0.14	1.98	7.55	0.79	1.99	0.48%
5	10 × 10 × 10	0.14	2.03				1.91%
6	13 × 13 × 13	0.14	2.05				2.71%
7	7 × 7 × 7	0.15	2.40	8.73	1.04	2.52	4.67%
8	10 × 10 × 10	0.15	2.45				2.65%
9	13 × 13 × 13	0.15	2.48				1.65%

**Table 4:** Comparison of a multi-layer BCC lattice sandwich structure and equivalent macro shear modulus between experiment, analysis and simulation.

**No**	**Structure Diameters D/mm**			**Structure Length L/mm**	**r/l**	$G_{\exp}/\mathrm{GPa}$	$G_{\mathrm{sim}}$ /Mpa	$G_{\mathrm{ana}}$ /Mpa	Error (Ana and Exp)
	**Min.**	**Max.**	**Aver.**						
1	1.17	1.23	1.2	3.464	0.173	3.65	3.50	3.58	1.89%
2	0.95	1.05	1.00	3.464	0.144	2.26	2.12	2.19	3.10%
3	0.93	1.04	0.985	3.464	0.142	2.19	2.04	2.09	4.6%
4	1.19	1.26	1.225	3.464	0.177	3.79	3.72	3.85	1.58%

## Data Availability

Data sharing is not applicable to this article.
